# Evaluation of Substrates of Al-Mg and Aluminized Steel Coated With Non-Stick Fluoropolymers after the Removal of the Coating

**DOI:** 10.3390/ma11112309

**Published:** 2018-11-17

**Authors:** Óscar Rodríguez-Alabanda, Pablo E. Romero, Guillermo Guerrero-Vaca

**Affiliations:** Department of Mechanical Engineering, University of Cordoba, Medina Azahara Avenue 5, 14071 Cordoba, Spain; orodriguez@uco.es (Ó.R.-A.); guillermo.guerrero@uco.es (G.G.-V.)

**Keywords:** Al-Mg, aluminized steel, fluoropolymers, coatings, removal

## Abstract

Many trays and pieces of Al-Mg and aluminized steel are used in the food industry. Sometimes these elements have non-stick coatings to solve problems related to the adhesion of masses and food products. With use, the coatings deteriorate and lose efficiency and must be removed to apply a new coating. The thermal cycles suffered by these alloys during the removal process of the deteriorated coating (500 °C) and the polymerization of a new coating (400 °C) can affect the durability and efficiency of the metallic substrates. The evolution of the mechanical and microstructural properties of the Al-Mg and aluminized steel substrates after two thermal cycles was studied in this work. The following parameters were analyzed: tensile strength, elongation (%), hardness, ASTM grain size, and the nature and distribution of the constituent particles. The report concluded that the removal of the coating, after each cycle, produced a decrease in the mechanical properties of the substrates. The hardness and tensile strength in Al-Mg decreases between 20–27% and in aluminized steel between 10–11%. In both cases, the process does not compromise the reuse of the substrate for the application of a new coating layer. The final blasting stage does not affect the Al-Mg alloys but may affect the aluminized steel Al-Si protective layer if special precautions are not taken.

## 1. Introduction

In the food industry, it is common to protect metal elements with non-stick coatings [1]. This is due to several reasons [2]: preventing food from sticking to substrates, facilitating cleaning tasks, facilitating demolding, and improving the quality of processed products. In addition, these coatings indirectly protect metal substrates and increase the service life of the elements.

Metal substrates used in the manufacture of trays, molds, and other food utensils must allow contact with food. Aluminum alloys, with certain precautions, meet this condition.

Common non-stick coatings include elastomers, sol-gel coatings (ceramics) in addition to some types of fluoropolymers [3]. Fluoropolymer coatings possess the following advantages [4]: high anti-adherence, high thermal stability, high chemical inertia, high scratch resistance, high hardness, high sliding angle, and good fixation to the substrate, among others.

When the substrates are exposed to a high number of uses (cycles), the coating loses properties: it wears, gets dirty and scratched, and degrades. In order to recover the support, it has to undergo a regeneration process, which basically consists of two stages: the first is a stripping process by thermal action [5] and the second is a sandblasting process [6].

### 1.1. Metals Substrates

The aluminum-magnesium alloys of the AW 5000 series are used intensively to produce trays, utensils, and molds for cooking ovens, among many other applications. They are highly regarded for their ability in cold deformation, weldability, mechanical resistance [7], and their excellent resistance to corrosion. The alloys EN-AW 5251 H34 and EN AW 5754 H32 are, moreover, thermally non-treatable and allow contact with food within certain limits [8,9,10,11].

With a similar objective, parts manufactured with aluminized steel are sometimes used. These are flat sheets of a hipoutectoid steel with a thin layer of aluminum-silicon on its two sides or faces. One example is the aluminized steel DX51D AS120 B CO according to the UNE-EN 10346 standard. This flat steel product combines the properties of aluminum, good corrosion resistance, and high thermal conductivity, with the excellent mechanical properties of steel [12]. To aluminize the steel substrate, a thin layer of an aluminum-silicon alloy [13] with a silicon content of 8–11% is fixed on the steel surface by a hot dip process. In these aluminized steel substrates, it is also possible to apply a non-stick coating with the same objectives previously mentioned. Both aluminum-magnesium and aluminized steel surfaces can be coated with fluoropolymers [14].

### 1.2. Fluoropolymer Coatings

Fluoropolymer coatings are especially suitable in the food industry due to their non-stick properties and chemical inertia [15]. Among the fluoropolymer resins used are those of polytetrafluoroethylene (PTFE), ethylene–propylene fluoride (FEP), perfluoroalkoxy copolymer (PFA), or a combination thereof [16]. These coatings are endowed with a very low surface energy [17].

Fluoropolymer coatings on metal substrates are formed by several layers. The layers are usually applied by spraying aqueous dispersions [18] and by electrodeposition of fluoropolymer powder particles [19]. The first layer serves to ensure a high bond to the metal substrate and allow subsequent anchoring of the second layer. The first layer, in this type of coating, should be dried in the oven at about 150 °C. The following layers should be polymerized between 390 and 425 °C, depending on the fluoropolymer [16].

### 1.3. Stripping

Coatings have a certain useful life, since, with use, they wear, scratch, degrade, and get dirty. At this point the deteriorated coatings are removed before applying a new coating. Parts and metal elements have, in many cases, a high value and, in this way, can be reused [20].

There are many industrial processes that are used in paint stripping, amongst them abrasive blasting [21], high pressure water jet [22], the use of plasma spraying [23], chemical procedures [24], cryogenic techniques [25], laser [26], high-density light pulses [27], pyrolysis, and certain combinations. Pyrolysis shows the highest rates for stripping [28] but needs to reach temperatures of 500 °C to ensure the degradation of the fluoropolymers.

In short, metallic substrates coated with PTFE, FEP, or PFA undergo various thermal cycles. Initially, drying at (150 °C), curing (390–425 °C) and afterward, the worn coating must undergo a removal of the coating at 500 °C. Finally, the parts are cleaned with sandblasting, very often with alumina particles, in order to eliminate any spent coating residue and prepare the surface for the new coating.

It is to be expected that in the end, the substrates may undergo some modifications in mechanical and superficial properties during their lifespan. Nevertheless, there have not been any studies found in the literature that tackle this problem.

### 1.4. Objetive of the Work

The objective of the present study is to evaluate the variation of the mechanical properties and microstructures of the materials commonly used in the industry of baking trays, utensils, and molds for bakery products. We focus on magnesium aluminum alloys, EN AW 5251 H34 [29], EN AW 5754 H32 [30] and aluminized steel DX51D AS120 B [31]. Modifications will be studied after two cycles of drying, curing-polymerizing, and removing the coating.

Additionally, it has been considered necessary to know the effects that may occur on the aluminium-silicon protective layer of the aluminized steel used in the study.

## 2. Materials and Methods

For the test program, 10 units of 140 mm × 120 mm were prepared for each alloy studied. The sheets were supplied by CAMEBE (Carpinteria Metalica Bengolea, Castro Urdiales, Spain). The aluminum-magnesium alloys (EN AW 5251 H34, EN AW 5754 H32) have a thickness of 1.2 mm and 1 mm for aluminized steel sheets (DX51D AS120 B).

The chemical composition of magnesium aluminum alloys and the aluminum–silicon layer of aluminized steel have been studied by X-ray microanalysis (EDX) JEOL JSM 6300 (JEOL, Peabody, MA, USA). The results are shown in Table 1 and are compatible with the standard composition.

**Figure 1:**
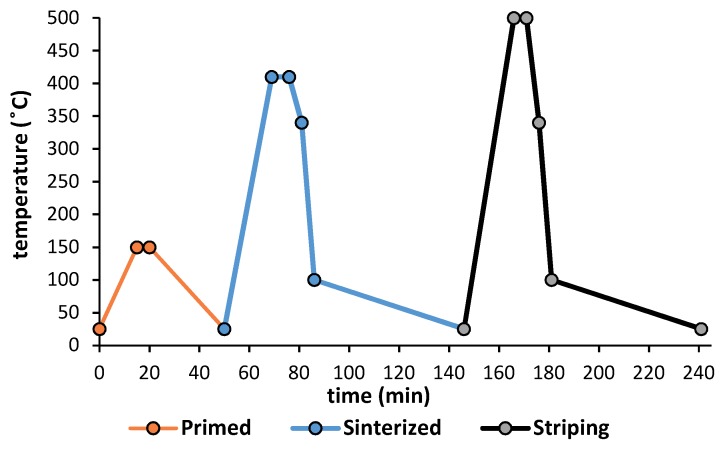
Thermal cycle 1 applied in substrates EN AW 5251, EN AW 5754, and DX51D AS120 B.

The thermal treatment consists of the drying of the primer layer, the curing-polymerization, and finally the removal of the coating. All this was carried out in a drying oven (up to 650 °C) with horizontal air circulation. The dimensions of the equipment were 295 mm wide, 340 mm deep and 170 mm high, with 2.7 kW of electrical power (NA 15/65, Nabertehem GmbH, Lilienthal, Germany). Figure 1 shows the thermal cycle to which the samples were exposed. These cycles are applied according to the recommendations of Whitford Company (Whitford Corporation, Elverson, PA, USA), a manufacturer of fluoropolymer resins (PTFE, FEP, and PFA).

### 2.1. Aluminum-Magnesium Alloys

For aluminum-magnesium substrates, metallographic samples EN-AW 5251 and EN-AW 5754 were prepared using cold-cured acrylic resin to obtain a polished colloidal silica finish. Thereafter, the Barker-type reagent (Struers, Madrid, Spain) was exposed (2 min, 20 V) electrolytically [32]. In all substrates, the microstructure was analyzed in a cross section and in another parallel to the direction of rolling, both in the reception and after the application and pickling stages of the coatings. Also characterized by a dispersive energy spectrometer (EDS) JEOL JSM 6300 (JEOL, Peabody, MA, USA) the main constituent particles (intermetallic) present in the alloys [33,34,35].

The Vickers hardness was obtained with a Zwick/Roell ZHU250 micro-durometer (Zwick Iberica Testing Equipment S.L, San Cugat del Valles, Barcelona, Spain) using a load of 300 g, according to UNE-ISO 6507 [36]. The tensile and elongation resistance was established with a 100 kN Zwick Roell Z100 electromechanical press in accordance with the standard, UNE-EN ISO 148-1. Flat traction specimens of standard type 1 were used. The results of the test of hardness and mechanical properties have been obtained as statistical mean values of 5 tests for each state.

### 2.2. Steel with Thin Layer of Aluminum–Silicon

To study the metallographic characteristics of the steel substrates, metallographic samples DX51D were prepared using hot cured resin with a polished colloidal silica finish. The samples were exposed to the reagent known as Marshall [37] (Struers, Madrid, Spain). Measurements of the ASTM grain size were made, for which grids were drawn with horizontal lines, over five different observation fields. The number of grains intercepted in a reference length was determined and the intercepted average length, $\bar{l}$ and grain size, *G*, was calculated [38].

Similar equipment to that used in aluminum-magnesium alloys has been used for the determination of Vickers hardness, tensile strength, and elongation.

The aluminum–silicon layer of the DX51D steel was studied and metallographic specimens were prepared, containing cross-sections of the material. The average thickness of the aluminum–silicon layer of the selected aluminized steel is 20 μm. Taking into account that according to the Al-Si diagram, for the maximum temperature range foreseen in the thermal cycle (500 °C), no phase transformations are to be expected [39], the mean diameter of the dendrites of the microconstituent α (Al) has been chosen as a possible parameter that can be influenced in the microstructure.

In addition, the influence of the projection treatment with abrasive particles has been studied in the thin aluminum–silicon layer. This is carried out after the stripping of coatings. The purpose of this projection is to clean the substrate of any previous residue and ensure a correct adhesion of the subsequent paint. A Sand Blast Cabinet CAT-990 (Aslak S.L, San Quirze del Valles, Barcelona, Spain) was used with an abrasive of the brown corundum type, RBT9 Gr.60 (Piedra Iberica, Madrid, Spain). The diameter of the projection nozzle was 6.5 mm, with a nozzle distance to the substrate of 200 mm and a pressure of 0.2 MPa. Projections were made in time intervals of 5, 10, and 15 s. The conditions of this test have been obtained by the data provided by Tecnimacor S.L (Villafranca de Cordoba, Cordoba, Spain), industry specialists in non-stick coatings.

## 3. Results

### 3.1. Aluminum-Magnesium Alloys

**Figure 2:**
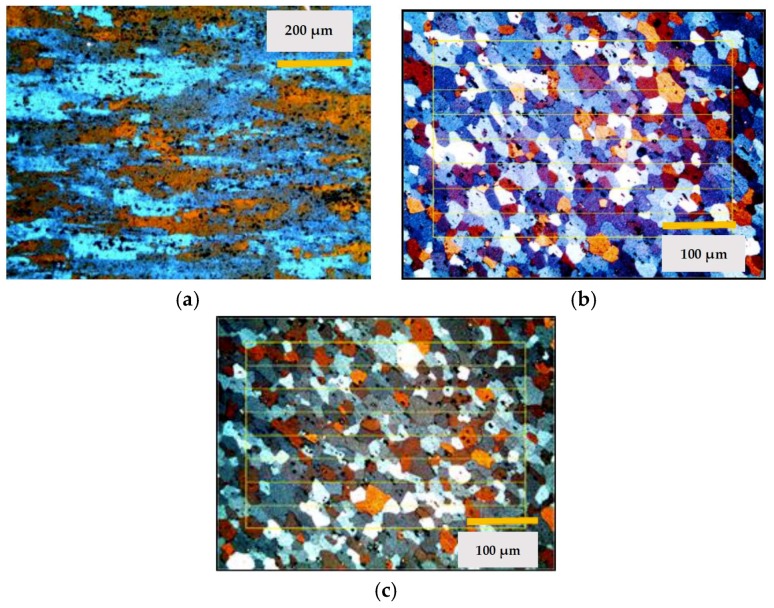
Metallographic images of EN-AW 5251, (**a**) microstructure in the supply state; (**b**) microstructure after thermal cycle 1; (**c**) microstructure after thermal cycle 2.

Micrographs were recorded of the aluminum in their supply state and after each cycle studied. The supply state of the alloy EN-AW 5251 is H34 which corresponds to a grade of ½ hard. Figure 2 shows the images of the states studied.

**Figure 3:**
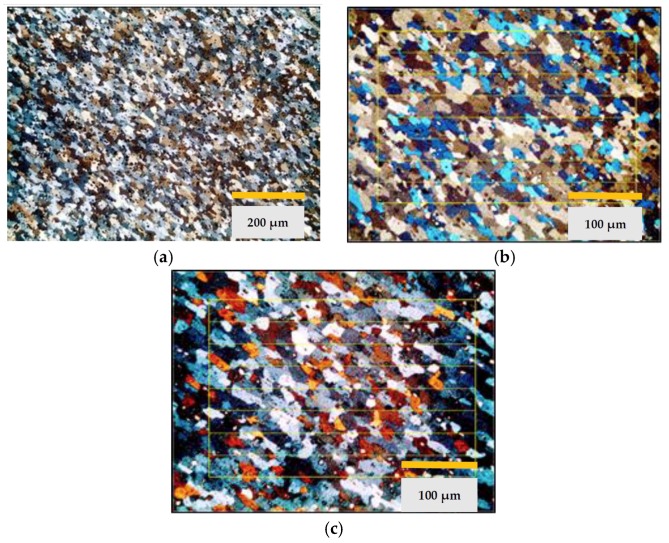
Metallographic images of EN-AW 5754, (**a**) microstructure in the supply state; (**b**) microstructure after thermal cycle 1; (**c**) microstructure after thermal cycle 2.

The images obtained of the alloy EN-AW 5754 are shown in Figure 3. In this case, the supply state was H32 which corresponds to ¼ hard.

**Figure 4:**
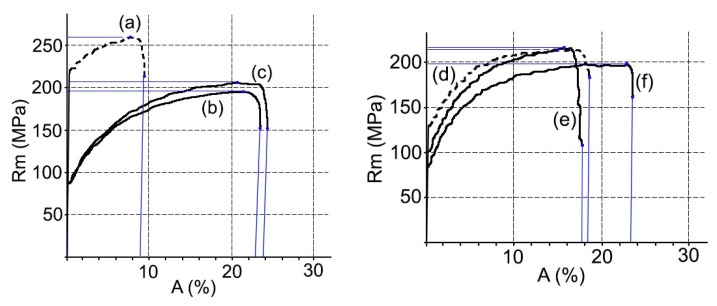
Stress–strain curves: AW 5241 supply (**a**), AW 5251 after cycle 1 (**b**), AW 5251 after cycle 2 (**c**), AW 5754 supply (**d**), AW 5754 after cycle 1 (**e**), and AW 5754 after cycle 2 (**f**).

At the same time, the mechanical properties, hardness, elongation, and tensile strength were studied, see Figure 4. On the other hand, the ASTM grain size and the fractions of area in % of the constituent particles that were characterized as Mg*x*Si and α-Al (Fe, Mn) Si. All results are shown in Table 2.

### 3.2. Steel with Thin Layer of Aluminum–Silicon

**Figure 5:**
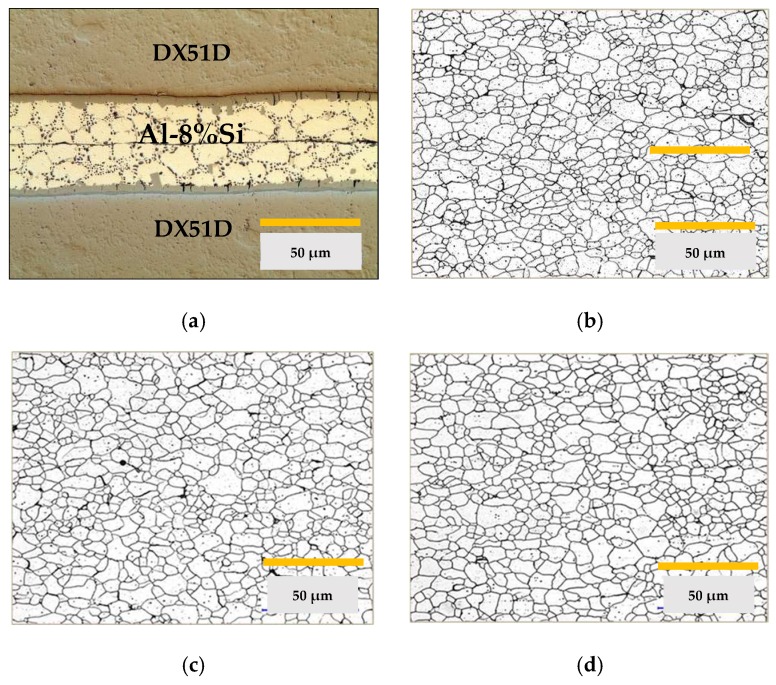
Metallographic images of DX51D steel, (**a**) detail of Al-Si coating in a set-up of sheets; (**b**) microstructure in supply state; (**c**) microstructure after thermal cycle 1; (**d**) microstructure after thermal cycle 2.

The metallographic images, see Figure 5, of the DX51D substrates were studied in the state of supply and after each of the two complete thermal cycles: drying of the base layer, polymerization-sintering of the final layer, and elimination of the coating. In the images, a totally ferritic structure is shown, typical of a steel with a very low carbon content in the annealed state.

**Figure 6:**
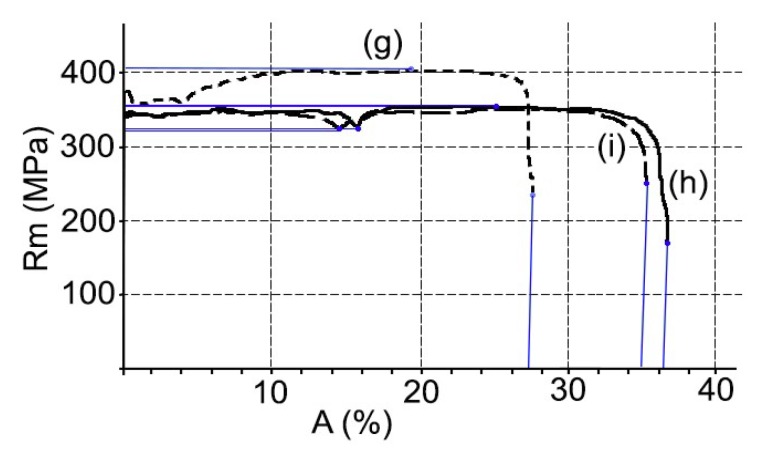
Stress–strain curve: DX51D supply (g); DX51D after cycle 1 (h); DX51D after cycle 2 (i).

The results obtained for the mechanical properties and microstructure are shown in Figure 6 and Table 3.

**Figure 7:**
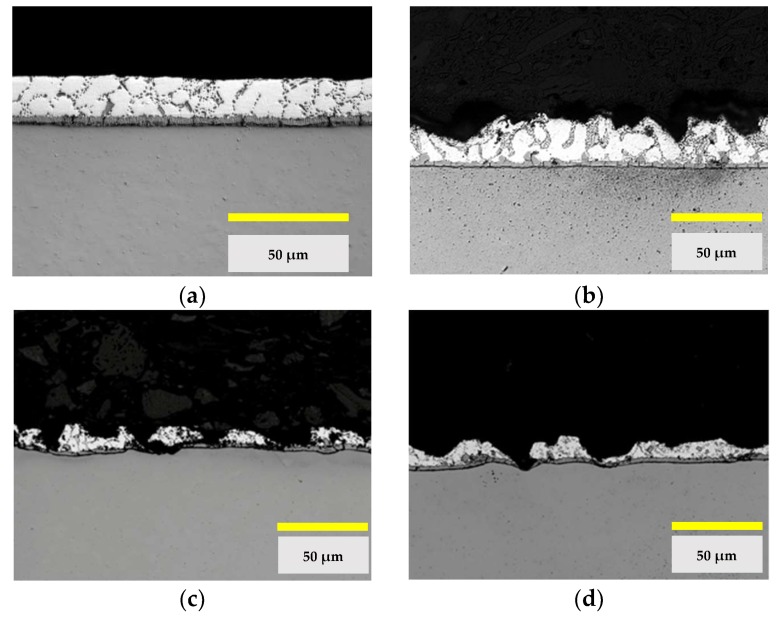
Micrographs of the Al-Si coating of the steel sheet DX1D + AS120, (**a**) state of supply; (**b**) after thermal cycle 2 and abrasive projection with brown corundum at 0.2 MPa for 5 s; (**c**) for 10 s and (**d**) for 15 s.

Finally, the aluminized steel substrates have been evaluated after the projection with abrasives using different time intervals at a pressure of 0.2 MPa. The results obtained are shown in Figure 7.

## 4. Discussion

In the case of aluminum alloy substrates EN-AW 5251 and EN-AW5457, the decrease in hardness and mechanical properties is mainly explained by the recrystallization that gave rise to the alloys after thermal cycle 1 [28]. In the recrystallized state, the variation in grain size between cycle 1 and cycle 2 is insignificant, as shown in the metallographic images and Table 2.

Changes in the mechanical properties between the supply state and the thermal cycles 1 and 2 are more intense in alloy EN AW 5251. Thus, the tensile strength decreases between 23–21%, the Vickers hardness between 27–26%, and the percentage elongation increases from 10.5% to 22–21%. In alloy AW 5754, the tensile strength decreases between 7.5–10%, the hardness Vickers 20–21%, and the percentage elongation goes from 18.7% to 20.1–23.1%.

However, after thermal cycles 1 and 2, the aluminum-magnesium alloys did not undergo significant changes in their microstructure or mechanical properties, as can be deduced from the values in Table 2. This may be due to the fact that there has been no modification in the size and distribution of the inter-metallic constituent particles. These particles have a high melting point and are not soluble in the temperature ranges used in the processes studied. In addition to this, the presence of dispersion particles of type Al (Fe, Mn), of submicroscopic size, act to block any movement of grain boundaries and, therefore, their growth [40,41].

In the case of the aluminized steel substrate DX51D + AS120 B CO, there is a decrease in Vickers hardness and tensile strength and an increase in the percentage of elongation, see Table 3. In summary, there has been a slight softening of the material between the states of supply and after each of the thermal cycles studied. This phenomenon is explained by a relaxation of the effect of the hardening by deformation that occurs in the rolling of the steel. In addition, in the order of the substructure, a decrease in dislocation density and, probably, a rearrangement, has been required [42]. This has produced a more stable structure and lower energy, without the microstructure being affected, as can be deduced from the discrete variation of their respective ASTM grain sizes [43].

Modifications to the mechanical properties of aluminized steel are more limited than those of aluminum-magnesium alloys. In this way, the tensile strength decreases by 12%, the Vickers hardness by 11%, and the percentage elongation increases from 27.9% to 34.2–36.3%.

Finally, the functional state of the Al-Si layer of the aluminized steel is studied after the thermal cycles and after the final cleaning of the substrate by abrasive projection. This stage has been applied to both aluminum-magnesium substrates and aluminized steel substrates. This is justified because it is necessary to remove the pyrolyzed coating and prepare the surface adequately for the subsequent coating. After the analysis of the micrographs of Figure 5, it is concluded that a severe degradation can occur which may compromise the continuity of the Al-Si layer. This point is relevant since it must be taken into account that the contact of the non-stick coating with the steel substrate without aluminum protection can cause contamination by oxidation, the loss of coating properties, and even the lack of fixing of the coating to the substrate.

The tests by means of projection with abrasives with corundum-brown particles show that at a pressure of 0.2 MPa and at a distance of 200 mm, the projection times greater than 5 s produce penetration of the steel substrate.

## 5. Conclusions

In the present work, the mechanical behavior of three materials (AW 5251, AW 5754, and DX51D + AS120) after being subjected to two thermal-mechanical cycles (primed-sintered-stripping-abrasive projection) has been evaluated. These cycles are similar to those carried out in coating and removal processes of metallic substrates with PTFE, FEP, or PFA.

Based on the results obtained, it can be affirmed that the substrates of aluminum-magnesium and aluminized steel permit successive stages of the application and elimination of coatings of PTFE, PFA, or FEP type and, therefore, can be reused for new coatings. In all cases, there have been variations in the mechanical and structural properties of the substrates. The variations allow the pieces to remain functional, although there has been appreciable softening in them.

Aluminized steel would be the best option if only the mechanical and structural properties were taken into account. However, the continuity of the Al-Si layer can be compromised after the projection by abrasive particles, and this fact can produce contamination by oxidation and can affect the coating. Under the conditions of the standardized test, it can be seen that the projection times of 5 s cannot be exceeded.

With regards to aluminum-magnesium alloys, AW 5754 is more suitable than AW 5215 alloy because its mechanical and structural properties are modified with less intensity. In this case, the substrates do not lose their functionality due to the projection of abrasive particles. The only effect is a slight increase in the roughness of the surface.

## Figures and Tables

**Table 1:** Chemical composition (wt.%) of aluminum-magnesium substrates and the aluminum-silicon layer of aluminized steel.

Substrates	Mg	Si	Fe	Mn	Cu	Zn	Cr	Ti	Al
EN AW 5251	1.9	0.15	0.4	0.5	0.1	0.3	0.02	0.01	rest
EN AW 5754	2.5	0.46	0.56	0.27	-	-	-	-	rest
Layer Al-Si AS120 B	0.01	8.3	0.45	0.1	0.05	-	-	-	rest

**Table 2:** ASTM grain size, tensile strength (R_m_), elongation rupture point (A_50_), Vickers hardness (HV) and % constituent particles on EN AW 5251 and EN AW 5754.

Alloy/State	Grain-Size (µm)/ASTM	R_m_ (MPa)	A_50_ (%)	HV 300 (g)	Area Fraction (%) α-Al (Fe, Mn) Si/Mg*_x_*Si
AW 5251/Supply	-	255.3 ± 2.2	10.5 ± 2.8	70.6 ± 2.1	2.52/0.14
AW 5251/After cycle 1	8.35 ± 0.28/11	196.1 ± 1.5	22.0 ± 1.3	52.1 ± 2.1	2.04/0.05
AW 5251/After l cycle 2	8.29 ± 0.32/11	202.1 ± 1.9	21.3 ± 2.2	51.0 ± 2.0	1.80/0.05
AW 5754/Supply	-	220.7 ± 2.4	18.7 ± 1.1	71.5 ± 1.8	2.58/0.19
AW 5754/After l cycle 1	8.51 ± 0.21/11	206.5 ± 1.0	20.1 ± 1.9	59.5 ± 2.2	2.49/0.28
AW 5754/After cycle 2	8.45 ± 0.26/11	200.6. ± 2.2	23.1 ± 2.0	58.1 ± 1.6	2.08/0.17

**Table 3:** ASTM grain-size, tensile strength (R_m_), elongation of rupture (A_50_), Vickers hardness (HV) of steel DX51D and average diameter (µm) of the micro-constituent dendrites α (Al) of the layer of Al-Si.

State	Grain-Size (µm)/ASTM	R_m_ (MPa)	A_50_ (%)	HV 300 (g)	Φ Average (µm) Dendrites α (Al)
Supply	11.33 ± 0.25/10	401 ± 3.6	27.9 ± 2.6	128 ± 1.7	10.26
After cycle 1	11.28 ± 0.15/10	353 ± 2.9	34.2 ± 3.1	114 ± 1.3	10.27
After cycle 2	11.23 ± 0.11/10	353 ± 4.1	36.3 ± 3.2	113 ± 1.9	11.07
